# PERSIANN-CCS-CDR, a 3-hourly 0.04° global precipitation climate data record for heavy precipitation studies

**DOI:** 10.1038/s41597-021-00940-9

**Published:** 2021-06-23

**Authors:** Mojtaba Sadeghi, Phu Nguyen, Matin Rahnamay Naeini, Kuolin Hsu, Dan Braithwaite, Soroosh Sorooshian

**Affiliations:** 1 Center for Hydrometeorology and Remote Sensing (CHRS), The Henry Samueli School of Engineering, Department of Civil and Environmental Engineering, University of California, Irvine, CA 92697 USA; 2 Department of Earth System Science, University of California Irvine, 3200 Croul Hall, Irvine, CA 92697-2175 USA

**Keywords:** Hydrology, Hydrology

## Abstract

Accurate long-term global precipitation estimates, especially for heavy precipitation rates, at fine spatial and temporal resolutions is vital for a wide variety of climatological studies. Most of the available operational precipitation estimation datasets provide either high spatial resolution with short-term duration estimates or lower spatial resolution with long-term duration estimates. Furthermore, previous research has stressed that most of the available satellite-based precipitation products show poor performance for capturing extreme events at high temporal resolution. Therefore, there is a need for a precipitation product that reliably detects heavy precipitation rates with fine spatiotemporal resolution and a longer period of record. Precipitation Estimation from Remotely Sensed Information using Artificial Neural Networks-Cloud Classification System-Climate Data Record (PERSIANN-CCS-CDR) is designed to address these limitations. This dataset provides precipitation estimates at 0.04° spatial and 3-hourly temporal resolutions from 1983 to present over the global domain of 60°S to 60°N. Evaluations of PERSIANN-CCS-CDR and PERSIANN-CDR against gauge and radar observations show the better performance of PERSIANN-CCS-CDR in representing the spatiotemporal resolution, magnitude, and spatial distribution patterns of precipitation, especially for extreme events.

## Abstract (summary)

**Table:** 

Measurement(s)	volume of hydrological precipitation • spatial resolution • spatial coverage • temporal resolution • temporal coverage
Technology Type(s)	digital curation
Sample Characteristic - Location	60°S - 60°N

Machine-accessible metadata file describing the reported data: 10.6084/m9.figshare.14656452

## Background & Summary

Precipitation is widely recognized as the main driving component for the global hydrological cycle and has an essential role for regulating the climate system^1–3^. Providing reliable estimation of precipitation, especially heavy precipitation, at fine spatial and temporal resolutions is crucial for many hydrological applications, including the development of water resources management and planning strategies, the development of early warning systems, as well as climatological studies^4–9^.

During the last three decades, a series of satellite-derived global precipitation datasets have been developed and made operational (for an overview, see Maggioni *et al*.^10^ Sun *et al*.^11^). Table 1 lists the spatiotemporal resolution and time coverage for the most widely used operational satellite-based precipitation products. These datasets vary from 0.04 to 2.5 degrees in terms of spatial resolution, from 30 minutes to monthly in terms of temporal resolution and up to 40 years of temporal span. These datasets have been designed for different applications and their usability depends upon the type of application along with the accuracy, latency, and temporal and spatial resolutions of the estimates.

**Table 1:** An overview of available operational satellite-based precipitation estimation products with their coverage and spatiotemporal resolutions.

Product	Full name	Spatial resolution	Spatial coverage	Temporal resolution	Temporal coverage	Reference
CHIRPS	Climate Hazards group Infrared Precipitation with Stations	0.05°	50°S–50°N	Daily	1981-present	^22^
CMAP	Climate Prediction Center (CPC) Merged Analysis of Precipitation	2.5°	90°S–90°N	Monthly/Pentad	1979-present	^49^
CMORPH	CPC morphing technique	0.25°	60°S–60°N	Daily	1998-present	^50^
GPCP	Global Precipitation Climatology Project (GPCP)	0.5°	90°S–90°N	Monthly/Pentad	1979-present	^33^
GPCP-1DD	Global Precipitation Climatology Project (GPCP) 1-Degree Daily (1DD) Combination	1°	90°S–90°N	Daily	1996–2015	^51^
IMERG	Integrated Multi-satellite Retrievals for GPM	0.1°	60°S–60°N	30 min	2014-present	^52^
MSWEP	Multi-Source Weighted-Ensemble Precipitation (MSWEP)	0.1°	90°S–90°N	3-hourly	1979–2017	^53,54^
PERSIANN	Precipitation Estimation from Remotely Sensed Information using Artificial Neural Networks	0.25°	60°S–60°N	1-hourly	2000–present	^55^
PERSIANN-CCS	PERSIANN-Cloud Classification System	0.04°	60°S–60°N	1-hourly	2003–present	^56^
PERSIANN-CDR	PERSIANN–Climate Data Record	0.25°	60°S–60°N	Daily	1983-present	^14^
**PERSIANN-CCS-CDR**	**PERSIANN-Cloud Classification System-Climate Data Record**	**0.04°**	**60°S–60°N**	**3-hourly**	**1983-present**	**This paper**
TMPA 3B42	TRMM Multi-satellite Precipitation Analysis	0.25°	50°S–50°N	3-hourly	1998-present	^57^

Climate studies applications require long-term precipitation records^12^; the World Meteorological Organization (WMO) reported that at least 30 years of weather information is needed for climatological studies^13,14^. Therefore, a reliable long-term global precipitation estimation product is crucial for climatological studies. On the other hand, several studies have shown that spatial–temporal variability of precipitation plays an important role in catchment response and performance of hydrological models. Therefore, developing a precipitation dataset with high spatial and temporal resolution is needed in order to represent urban runoff processes well^15–20^. For example, Lobligeois *et al*.^15^ investigated the impact of the spatial resolution of precipitation inputs on the performance of semi-distributed hydrological models over 181 catchments with a variety of sizes and climate conditions. They concluded that semi-distributed models significantly outperform lumped models when precipitation inputs have higher spatial resolution, while they perform similarly when precipitation have low spatial resolution. Huang *et al*.^17^ explored the sensitivity of hydrological model performance to the temporal resolution of precipitation inputs using lumped and distributed models. They showed that providing sub daily precipitation estimates rather than daily inputs can significantly improve the performance of the hydrologic model.

Nonetheless, most of the available operational precipitation products are either high resolution/short-duration estimates or low resolution with long-term estimates^21^; therefore, there is a need for providing precipitation products with both fine spatial and temporal resolution and a long period of record. Precipitation products with such attributes would provide the opportunity for researchers to study the spatial details and complete evolutions of extreme events including hurricanes and convective storms in the context of historical events^22–24^. Most of the currently available satellite-based precipitation estimation products are not ideal for detecting extreme events at high altitude^25^ as well as at high temporal resolutions (3-hourly)^6,26–29^. For example, Mehran *et al*. (2014) evaluated the performance of Tropical Rainfall Measuring Mission (TRMM) Multisatellite Precipitation Analysis (TMPA), Precipitation Estimation from Remotely Sensed Information Using Artificial Neural Networks (PERSIANN) and CPC MORPHing technique (CMORPH) for detecting heavy precipitation rates against Stage IV radar observations over the CONUS. They showed that all these precipitation datasets miss a significant volume of rainfall. They concluded that none of the 3-hourly estimates from these products are suitable for detecting extreme events and their detection skills decrease dramatically as the extreme threshold increases. Precipitation Estimation from Remotely Sensed Information using Artificial Neural Networks-Cloud Classification System-Climate Data Record (PERSIANN-CCS-CDR), which provides 0.04° spatial and 3-hourly temporal resolution estimates from 1983 to present, has been explicitly designed to address the need for having a long term dataset with fine spatiotemporal resolution precipitation estimation which is reliable for extreme event detection.

## Methods

### Input data

#### Gridded satellite infrared data (GridSat-B1)

In 1983, the National Oceanic and Atmospheric Administration (NOAA)/National Climatic Data Center (NCDC) began collecting meteorological geostationary satellite data through the International Satellite Cloud Climatology Project (ISCCP)^30^. The ISCCP B1 dataset provides global IR brightness temperature data with 10-km spatial and 3-hourly temporal resolution for the period from 1979 to present. The ISCCP B1 dataset consists of observations from different sensors launched by different countries, including United States [for the Geostationary Operational Environmental Satellite (GOES) series], Japan [for the Japanese Geostationary Meteorological Satellite (GMS) series and Multi-functional Transport Satellite (MTSAT)], Europe [for the European Meteorological satellite (Meteosat) series], and China [for the Chinese Fen-Yung 2 (FY2) series]. The gridded satellite (GridSat-B1) dataset isderived from the ISCCP B1 dataset and provides near-global data with 0.07° spatial and 3-hourly temporal resolution from 1980 to present. The GridSat-B1 dataset is available via (https://www.ncdc.noaa.gov/gridsat/gridsat-index.php). More information can be found in Knapp *et al*.^31^.

#### NOAA Climate Prediction Center (CPC-4km) IR product

The NOAA Climate Prediction Center (CPC) globally merged IR product, which is referred to as CPC-4km, was developed to provide near-real time data for monitoring global precipitation^32^. This dataset offers near-global (60°N–60°S) IR data with 4-km spatial and half-hourly temporal resolutions from the present international constellation of operational geostationary meteorological satellites for the period from 2000 to present. The CPC-4km product is comprised of IR observations from several international GEO satellites, including Meteosat-5 and Meteosat-7, GMS, and GOES. CPC-4km data is publicly accessible through the Climate Prediction Center webpage (https://www.cpc.ncep.noaa.gov/products/global_precip/html/web.shtml).

#### Global Precipitation Climatology Project (GPCP V2.3)

GPCP is part of the Global Energy and Water Cycle Exchanges (GEWEX) project under the World Climate Research Program (WCRP)^33^. This product provides monthly precipitation at a 2.5° × 2.5° spatial resolution by merging different satellite-based estimation information (passive microwave/infrared) along with precipitation gauge networks from GPCC. A comprehensive description of GPCP monthly v2.3 data inputs and the merging process can be found in^34^. The GPCP dataset is available for public use through the Earth System Science Interdisciplinary Center (ESSIC) and Cooperative Institute for Climate and Satellites (CICS), University of Maryland College Park (http://gpcp.umd.edu).

### Reference data

#### CPC Global unified gauge-based analysis of daily precipitation

The National Oceanic and Atmospheric Administration (NOAA) Climate Prediction Center (CPC) provides the CPC Global Unified Gauge-Based Analysis of Daily Precipitation dataset^35^. This dataset offers global precipitation estimates at 0.5° × 0.5° spatial and daily temporal resolutions from 1979 to present. The daily precipitation reports of roughly 30,000 stations from different sources across global land areas have been collected and quality controlled by the NOAA Climate Prediction Center. The data sources include Global Telecommunication System (GTS), the CPC unified daily gauge datasets over the CONUS, Cooperative Observer Network (COOP), and other national and international agencies. A comprehensive description of this CPC dataset and the interpolation algorithm that is used can be found in Xie *et al*.^36^. The CPC Global Unified Gauge-Based Analysis of Daily Precipitation dataset is accessible to the public through (ftp://ftp.cdc.noaa.gov/Datasets).

#### NCEP Stage IV QPE Data

NCEP Stage IV QPE is widely considered as the best long-term gridded rain accumulation dataset over the CONUS due to its extensive quality control procedures^6,37^. This product merges the national Weather Surveillance Radar-1988 Doppler (WSR-88D) network of ground radars and ground-based rain gauge observations^38,39^. NCEP Stage IV provides precipitation observations at 0.04° (4 km) spatial resolution and hourly, 6 hourly, and 24 hourly temporal resolution. For this study, daily NCEP Stage IV observations were obtained from the distribution website (http://www.emc.ncep.noaa.gov/mmb/ylin/pcpanl/stage4/) and used as the benchmark for evaluating the remotely sensed precipitation datasets over the CONUS.

#### Precipitation Estimation from Remotely Sensed Information using Artificial Neural Networks–Climate Data Record (PERSIANN-CDR)

PERSIANN-CDR is a satellite-based precipitation estimation product that provides more than three decades (from 1983 to present) of daily precipitation estimates at 0.25° × 0.25° spatial resolution for the 60°S–60°N latitude band^14^. PERSIANN-CDR utilizes the archive of infrared brightness temperature from GridSat-B1^30,40^ as the input of the PERSIANN algorithm. Then the rainfall estimates of the PERSIANN algorithm are bias corrected using the monthly Global Precipitation Climatology Project (GPCP) version 2.3 product at 2.5° × 2.5° spatial resolution^41^. This dataset is available for public access through the NOAA National Centers for Environmental Information (NCEI) Program (https://www.ncdc.noaa.gov/cdr) and through the Center for Hydrometeorology and Remote Sensing (CHRS) Data Portal (http://chrsdata.eng.uci.edu/). Additional details about the PERSIANN-CDR algorithm can be found in Ashouri *et al*.^14^ and Sadeghi *et al*.^41–43^.

#### PERSIANN-CCS-CDR Methodology description

PERSIANN-CCS-CDR is generated beginning with rain rate (RR) outputs from the PERSIANN-CCS model. It should be noted that the PERSIANN-CCS algorithm utilizes one global IR image to estimate the rainfall for that corresponding time step. The IR inputs used by PERSIANN-CCS to generate RR outputs come from two distinct periods: From 1983 through February 2000 GridSat-B1 IR data are used but from March 2000 to the present CPC-4km IR data are available and those are used due to its higher quality. It should be mentioned that we observed some inconsistencies between PERSIANN-CCS-CDR constructed using GridSat-B1 IR and CPC-4KM IR; however, they results were consistence for most of the globe. All IR inputs are resampled to 0.04° resolution before input. The GridSat-B1 images are every 3 hours and the output RR data are mm/hr rain rates every 3 hours. The input CPC-4km images are available every 30-minutes, so the output RR data are mm/hr rain rates every 30-minutes.

In the second stage of the PERSIANN-CCS-CDR model the 0.04° RR grids, after a threshold (thd) of 0.1 mm/3 hr is applied, are aggregated to monthly temporal resolution for comparison to monthly GPCP v2.3 precipitation.Due to the nature of satellite IR data, some fractions of non-raining pixels are falsely associated with light precipitation in the PERSIANN-CCS-CDR algorithm. We applied a threshold (thd) of 0.1 mm/hr to eliminate these falsely assigned light rain rate values. The monthly PERSIANN-CCS RR accumulations (mm/month) at 0.04° spatial resolution must be aggregated then to 2.5° using the bilinear method to match GPCP v2.3.

1 $${{\rm{R}}}_{{\rm{Cum}}-{\rm{PERSIANN}}-{\rm{CCS}}}({\rm{i}}{\prime} ,{\rm{j}}{\prime} )=\sum ^{{\rm{nd}}}\sum ^{{\rm{nh}}}\left(\sum ^{{\rm{62}}}\sum ^{{\rm{62}}}\left[{{\rm{r}}}_{{\rm{PERSIANN}}-{\rm{CCS}}}({\rm{i}},{\rm{j}})\ge {\rm{thd}}\right]\right)$$

In this equation, ${{\rm{R}}}_{{\rm{Cum}}-{\rm{PERSIANN}}-{\rm{CCS}}}$ and ${{\rm{r}}}_{{\rm{PERSIANN}}-{\rm{CCS}}}$ are the monthly 2.5° aggregated PERSIANN-CCS estimates and 30 min/3-hourly PERSIANN-CCS estimates at 0.04° spatial resolution, respectively. The i and j represent the latitude and longitude of the 30 min/3-hourly PERSIANN-CCS at 0.04° × 0.04° spatial resolution. Similarly, i′ and j’ are the latitude and longitude of the aggregated PERSIANN-CCS at 2.5° × 2.5° resolution. The nd and nh are the number of days and the number of 30 min/3-hourly PERSIANN-CCS samples in each day, respectively.

In the next stage, the bias-adjustment weights for each monthly 2.5° grid cell are calculated as follows:

2 $${\rm{w}}\left({\rm{i}}{\prime} ,{\rm{j}}{\prime} \right)={{\rm{R}}}_{{\rm{GPCP}}}\left({\rm{i}}{\prime} ,{\rm{j}}{\prime} \right)/{{\rm{R}}}_{{\rm{Cum}}-{\rm{PERSIANN}}-{\rm{CCS}}}\left({\rm{i}}{\prime} ,{\rm{j}}{\prime} \right),0\le {\rm{w}}\le 2.5,$$

where R_GPCP_ is the monthly rain rate of GPCP for a given pixel.

The 2.5° weights grids must then be bilinearly interpolated back to 0.04° spatial resolution for the next stage. Our experiments showed that some locations with very low monthly rainfall values, such as high latitudes and very dry regions, the weight (w) can become large. This can lead to unreasonably large 3-hourly rainfalls at finer resolution. To prevent such cases, we applied a maximum weight of 2.5. In the final 2 stages, the 0.04° monthly weights are applied to each original input RR grid available. Each RR grid, either every 3 hours RR or every 30-minute RR depending on the IR data source, is multiplied by the weight.

3 $${\rm{PERSIANN}}-{\rm{CCS}}-{\rm{CDR}}({\rm{i}},{\rm{j}})={\rm{w}}({\rm{i}},{\rm{j}})\ast {{\rm{r}}}_{{\rm{PERSIANN}}-{\rm{CCS}}}({\rm{i}},{\rm{j}})$$

Finally, the bias-adjusted RR files are aggregated to produce the final PERSIANN-CCS-CDR product, consisting of an accumulation at 3-hourly resolution and 0.04° spatial resolution. Due to the limitation of IR information and precipitation algorithm, the RR mm/hr are limited by a historical maximum rain rate for each local region and each calendar month for seasonal and geographical specificity. We monitor this threshold over the time, and we will adjust this threshold to consider the climate change.

## Data Records

A near-global 37+ year high-resolution precipitation dataset with high spatial and temporal resolution is now available. PERSIANN-CCS-CDR developed by the Center for Hydrometeorology and Remote Sensing (CHRS) at the University of California, Irvine (UCI) provides precipitation estimates at 0.04° spatial and 3-hourly temporal resolutions from 1983 to present over the global domain of 60°S to 60°N. PERSIANN-CCS-CDR data are publicly available through CHRS Data Portal (https://chrsdata.eng.uci.edu/) and also on our FTP site (ftp://persiann.eng.uci.edu/CHRSdata/PCCSCDR/) for a number of time steps (3-hourly, 6-hourly, and daily), and different formats (NetCDF, GeoTiff, and Esri BIL) from 1983 to present. The detailed description of the data format can be found in the provided FTP link^44^.

## Technical Validation

### Performance Evaluation for extreme events over the Globe and the CONUS

**Fig. 1:**
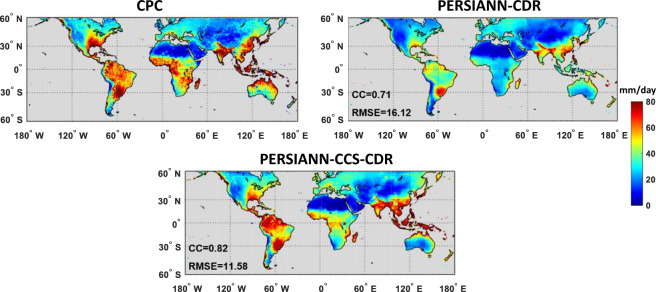
Evaluation of PERSIANN-CDR and PERSIANN-CCS-CDR against CPC for the 99th percentile of daily precipitation on wet days (days with daily precipitation > = 1 mm) for the period of 1983–2019.

Figure 1 presents the performance of PERSIANN-CDR and PERSIANN-CCS-CDR against CPC in detecting the 99^th^ percentile indices (RR99p) of the daily precipitation on wet days (days with daily precipitation > = 1 mm) for the period of 1983 to 2019 over global land areas. The CPC unified gauge-based analysis dataset at 0.5° × 0.5° spatial resolution is used as a gauged-based reference for comparing and calculating the continuous evaluation indices including CC, RMSE, and MSE. The original 0.25° PERSIANN-CDR and 0.04° PERSIANN-CCS-CDR datasets are resampled to 0.5° spatial resolution using the bilinear interpolation method to match the spatial resolution of the CPC dataset. Then, the 99th percentile of daily precipitation of CPC, PERSIANN-CDR, and PERSIANN-CCS-CDR datasets were calculated for each pixel using the whole period of record (1983–2019). Clearly in Fig. 1, PERSIANN-CCS-CDR offers more accuracy for the intensity of rain rates in comparison to PERSIANN-CDR in terms of estimating extreme events. PERSIANN-CDR tends to underestimate heavy precipitation over the globe, while PERSIANN-CCS-CDR provides a more realistic representation of heavy precipitation. Figure 1 also reveals that PERSIANN-CCS-CDR’s estimates have both higher correlation and lower RMSE with CPC observations compared to PERSIANN-CDR over the globe. The correlation between PERSIANN-CCS-CDR and CPC unified gauge-based analysis over land is increased by 15%, and the RMSE decreased by 28%, respectively, versus PERSIANN-CDR.

**Fig. 2:**
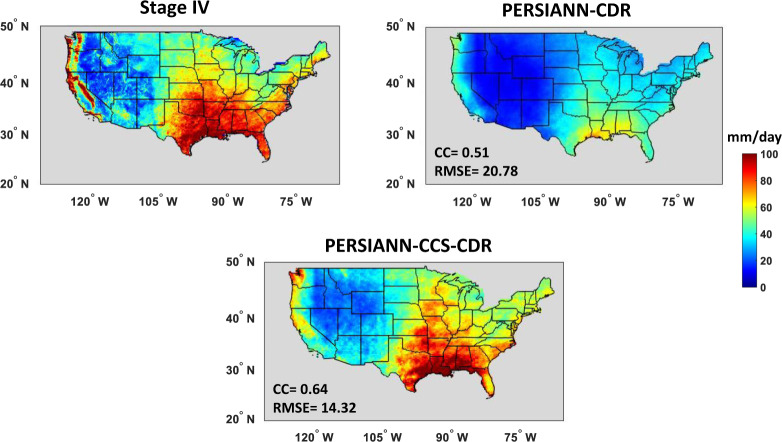
Evaluation of PERSIANN-CDR and PERSIANN-CCS-CDR against Stage IV for the 99th percentile of daily precipitation on wet days (days with daily precipitation > = 1 mm) for the period of 2003–2019.

Figure 2 shows the performance of PERSIANN-CDR and PERSIANN-CCS-CDR against Stage IV, as a reference, in capturing the RR99p of daily precipitation at 0.25° × 0.25° spatial resolution for the period of 2003 to 2019. We used daily Stage IV images, which depict 24-hour total precipitation ending at 1200 UTC. In addition, we utilized the PERSIANN-CDR and PERSIANN-CCS-CDR estimates with the same end of day definition. The PERSIANN-CCS-CDR estimates were resampled to 0.25° spatial and daily temporal resolution using the bilinear method for the comparison. As shown in Fig. 2, high values of RR99p appear in the southeastern part of the United States. In general, PERSIANN-CCS-CDR captures the magnitude and the pattern of RR99p better than PERSIANN-CDR compared to radar observations, especially over the southeastern part of the CONUS. Although PERSIANN-CDR shows similar patterns to Stage IV, it underestimates magnitudes of precipitation. On the other hand, PERSIANN-CCS-CDR captures the volume of the extreme events fairly well. The disagreement between PERSIANN-CCS-CDR and Stage IV radar observations is mainly over the northern part of the United States, including Minnesota, Iowa, and Michigan, where most values are overestimated. According to Fig. 2, the spatial correlation, RMSE, and MSE of PERSIANN-CDR and PERSIANN-CCS-CDR with respect to CPC unified gauge-based analysis indicate that PERSIANN-CCS-CDR has better performance than PERSIANN-CDR for extreme event analyses in climatological applications. CC and RMSE of PERSIANN-CCS-CDR estimates with Stage IV are improved by 25% and 31%, in comparison to PERSIANN-CDR, respectively. PERSIANN-CCS is an improvement of the PERSIANN algorithm; Instead of direct pixel-to-pixel fitting of IR cloud top temperature to the rain rate, PERSIANN-CCS leverages objective classification methods in order to classify each IR image into a number of cloud patch groups based on feature similarity including patch size and texture. This feature results in higher performance of PERSIANN-CCS compared to PERSIANN in capturing extreme precipitation. Utilizing PERSIANN-CCS estimates as inputs to PERSIANN-CCS-CDR leads to improved performance in capturing extreme precipitation compared to PERSIANN-CDR, which uses PERSIANN estimates.

### Case studies

To further investigate the performance of PERSIANN-CCS-CDR estimates with Stage IV as a reference, the following verification studies have been done at different temporal and spatial resolutions. The first case study targets Hurricane Harvey in 2017 and investigates the performance of the PERSIANN-CCS-CDR estimates over the Southeastern United States at daily temporal resolution. The second case study is related to the flood that occurred in Louisiana in 2016 and evaluates the performance of the developed product at the watershed scale for 3-hourly and daily temporal scales.

### Hurricane Harvey, August 2017 (Daily Assessment)

**Fig. 3:**
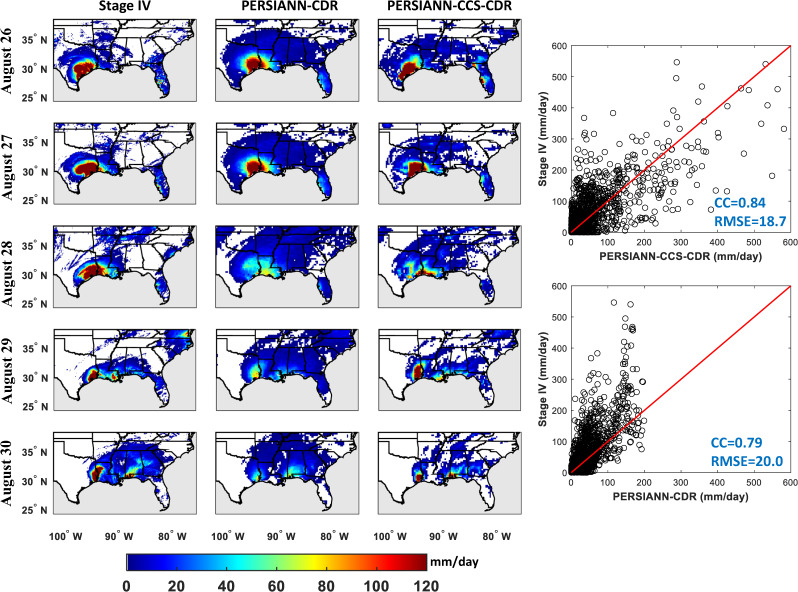
The spatial precipitation evolution of Hurricane Harvey for the period from August 26–30, 2017 from (**a**) Stage IV, (**b**) PERSIANN-CDR, and (**c**) PERSIANN-CCS-CDR.

During August 25–30, 2017, Hurricane Harvey hit the Southeastern regions of the United States, including Southeast Texas, and Louisiana. Hurricane Harvey is classified as a Category 4 storm that caused catastrophic damages. This storm is referred as one of the costliest extreme precipitation events that struck in the history of the United States^45^. According to the National Hurricane Center, the total damage is estimated to be more than $125 billion and it is confirmed that there were more than 80 fatalities^46^. In this section, PERSIANN-CCS-CDR and PERSIANN-CDR are evaluated against Stage IV as the reference at 0.25° spatial resolution and daily temporal resolution. The original 0.04° × 0.04° spatial resolution of Stage IV and PERSIANN-CCS-CDR datasets were resampled to 0.25° × 0.25° spatial resolution using bilinear interpolation to match the spatial resolution of PERSIANN-CDR. Then the 3-hourly estimates of PERSIANN-CCS-CDR were aggregated to obtain daily scale to match the PERSIANN-CDR estimates in temporal resolution. Figure 3 presents daily values for extreme precipitation that occurred from August 27 to 30, 2017 using Stage IV data and PERSIANN-CDR and PERSIANN-CCS-CDR estimates. As shown in Fig. 3, PERSIANN-CCS-CDR captures both the spatial pattern and the intensity of rainfall better than PERSIANN-CDR. For more exploration of the accuracy of estimation, the scatter plots for PERSIANN-CDR and PERSIANN-CCS-CDR versus Stage IV are presented and the relevant statistics are calculated in Fig. 3. In general, PERSIANN-CCS-CDR outperforms PERSIANN-CDR for CC and FAR. As shown in Fig. 3, both PERSIANN-CDR and PERSIANN-CCS-CDR show a high correlation with Stage IV radar. The correlation for PERSIANN-CDR and PERSIANN-CCS-CDR with Stage IV are 0.79 and 0.84, respectively. RMSE improves from 20 mm/day to 18.7 mm/day for PERSIANN-CCS-CDR estimates compared to PERSIANN-CDR. Furthermore, PERSIANN-CDR underestimates intense precipitation, while PERSIANN-CCS-CDR performs relatively well. As can be seen, both Stage IV and PERSIANN-CCS-CDR show rain rates with more than 500 mm/day that occurred over some pixels. However, PERSIANN-CDR does not show any value more than 195 mm/day and underestimates the rain rates.

### Louisiana Flood, August 12, 2016 (3-hourly, and Daily Assessment)

**Fig. 4:**
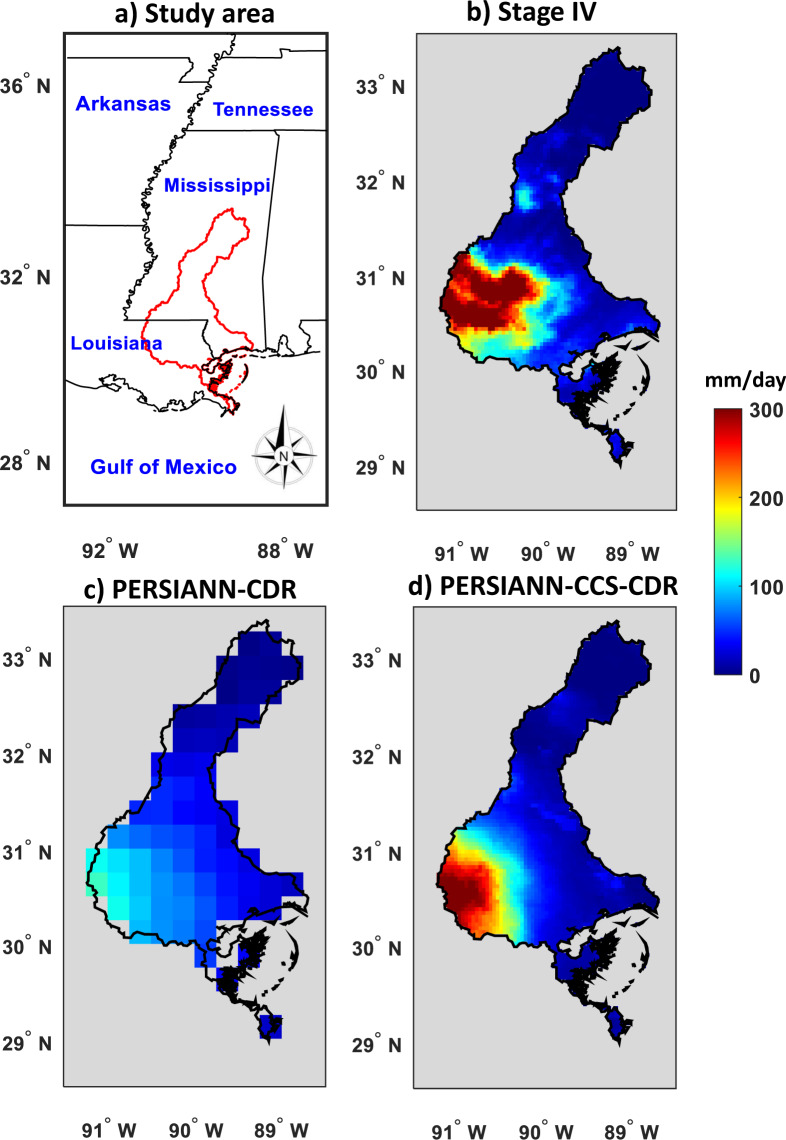
Evaluation of daily PERSIANN-CDR and PERSIANN-CCS-CDR against Stage IV for the Louisiana flood of Aug. 12, 2016.

In August 2016, an intense rainfall event occurred over the state of Louisiana with more than 30 inches (760 millimeters) of rainfall in some locations, resulting in a catastrophic flood (https://www.usgs.gov/news/). This event led to more than a dozen deaths and more than $30 million in damages (https://www.cnn.com/). The performances of PERSIANN-CDR and PERSIANN-CCS-CDR for this flood are evaluated against Stage IV radar observations (Fig. 4). Figure 4b shows Stage IV rainfall estimates at 0.04° × 0.04° spatial resolution on August 12, 2016. Figure 4c,d shows PERSIANN-CDR estimates (with a 0.25° × 0.25° spatial resolution) and PERSIANN-CCS-CDR estimates (with a 0.04° × 0.04° spatial resolution), respectively. This figure demonstrates two attractive aspects of the PERSIANN-CCS-CDR dataset in comparison to the PERSIANN-CDR dataset for climatological studies: 1) PERSIANN-CCS-CDR performs better than PERSIANN-CDR for this extreme event by capturing the volume of heavy rain over the southwest area of the watershed as shown. 2) The high temporal resolution of PERSIANN-CCS-CDR is another feature which is beneficial for studying the diurnal cycle and is essential for rainfall-runoff modeling studies. These two features of PERSIANN-CCS-CDR make this new dataset attractive for integrating its high spatiotemporal resolution estimates into hydrological and land-surface models for flood forecasting (or other applications that are sensitive to heavy precipitation rates).

**Fig. 5:**
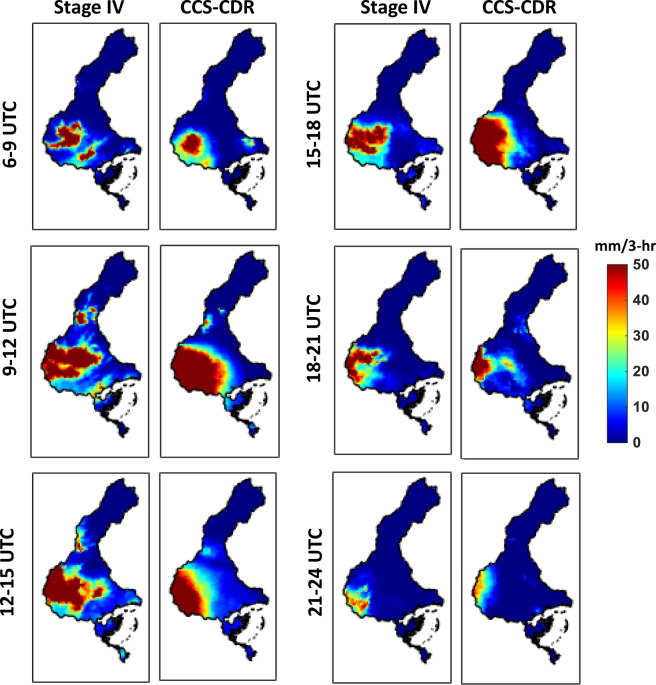
Evaluation of 3-hourly PERSIANN-CCS-CDR against Stage IV for the Louisiana flood of Aug. 12, 2016.

**Fig. 6:**
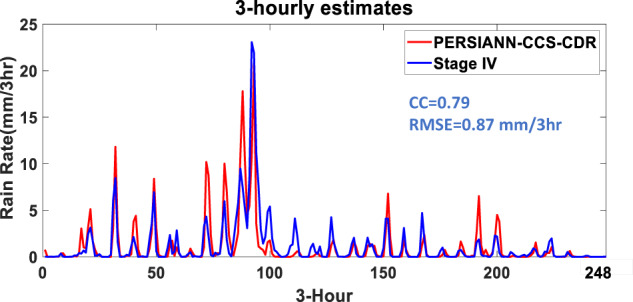
3-hourly estimates of Stage IV and PERSIANN-CCS-CDR over the Louisiana watershed shown in Fig. 5 for the month of August, 2016.

Figure 5 illustrates the evolution of the precipitation that occurred on August 12, 2016 based on Stage IV and PERSIANN-CCS-CDR estimates at 3-hourly temporal and 0.04° × 0.04° spatial resolutions. The hourly estimates of Stage IV data are aggregated to a 3-hourly scale to match the PERSIANN-CCS-CDR data in temporal resolution. This figure demonstrates the superior performance of PERSIANN-CCS-CDR in detecting the magnitude of precipitation at the 3-hourly temporal scale. For the period between 6:00 to 9:00 UTC, PERSIANN-CCS-CDR estimates the rain rates fairly well compared to Stage IV radar data; however, a southward shifting can be seen in PERSIANN-CCS-CDR’s estimates. During 9:00 to 12:00 UTC, 12:00 to 15:00 and 15:00 to 18:00 UTC, PERSIANN-CCS-CDR successfully detects the amount of intense precipitation over the eastern regions of the watershed, yet it misses some parts of the rainfall that occurred over the central parts of the region. During 18:00 to 21:00 UTC, the PERSIANN-CCS-CDR detects most of the intense rainfall. A northward shift can be seen in PERSIANN-CCS-CDR’s estimates for the period of 21:00 to 24:00 UTC. Table 2 summarizes PERSIANN-CCS-CDR performance in detecting (POD, FAR, CSI) and estimating (RMSE, MAE, Correlation) rainfall intensity at 3-hourly temporal and 0.04° × 0.04° spatial resolutions over the shown watershed on August 12, 2016. Figure 6 shows the 3-hourly estimates of Stage IV (blue) and PERSIANN-CCS-CDR (red) over the Louisiana watershed shown in Fig. 5 for the month of August 2016. It can be seen that PERSIANN-CCS-CDR can capture both the pattern and the 3-hourly peak precipitation relatively well compared to Stage IV as the reference. Specifically, the CC and RMSE of PERSIANN-CCS-CDR with Stage IV are 0.79 and 0.87 mm/3 hr, respectively.

**Table 2:** Summary of 3-hourly precipitation estimation performance of PERSIANN-CCS-CDR for the Louisiana flood of Aug.

Criteria	PERSIANN-CCS-CDR
POD	0.85
FAR	0.24
CSI	0.67
RMSE (mm/3 hr)	13.6
MAE (mm/3 hr)	6.12
CC	0.64

12, 2016 at 0.04° × 0.04° spatial resolution.

One of the most attractive features of PERSIANN-CCS-CDR is its improved performance for detecting heavy precipitation. While current operational satellite-based precipitation datasets provide significant opportunities for providing precipitation estimates at high spatiotemporal resolution over the globe, numerous studies have argued that most of them have a weakness in detecting heavy precipitation that occurs at high temporal resolution (sub-daily or 3-hourly)^6,26^. The current limitations for accurate estimation of extreme precipitation hinder the use of satellite-based precipitation datasets for applications that are sensitive to heavy rain rates such as designing warning systems, integrating precipitation data into hydrological models, etc. The performance of PERSIANN-CCS-CDR for heavy precipitation detection suggests that this dataset is an attractive dataset for the mentioned hydrological applications.

Another important feature of the PERSIANN-CCS-CDR dataset is the combination of high spatial and temporal resolution with a long period of record. A reliable long-term precipitation record with fine spatiotemporal resolution is essential for many applications including hydrological modelling, rainfall frequency analysis, and development of depth duration curves. However, the majority of current operational datasets are either high resolution with short-duration estimates or lower resolution with long-term estimates^21^. PERSIANN-CCS-CDR, which provides accurate estimations of extreme precipitation with fine spatiotemporal resolution (0.04° × 0.04° spatial and 3-hourly temporal resolution) from 1983 to present over the global domain of 60°S to 60°N, has been developed to address the lack of available datasets that meet both needs.

One of the few potential shortcomings of the PERSIANN-CCS-CDR dataset is that the available time scale of GridSat-B1 is 3-hourly, rather than hourly, for the period before 2000. This limits the capability of the dataset to that coarser temporal resolution for the period from 1983 to 2000. Another limitation is that PERSIANN-CCS, as an IR-based precipitation estimation algorithm, link the IR cloud-top temperature (brightness) to the probability and intensity of rainfall. PERSIANN-CCS utilizes 253 Kelvin cloud temperature as the threshold for patch feature extraction. This threshold can limit the capability of PERSIANN-CCS in detection and estimation of warm rainfall. Another limitation is the PERSIANN-CCS algorithm’s capability to capture the spatial patterns of rainfall. PERSIANN-CCS extracts cloud information based on manually defined features including coldness, texture, and geometry, which can limit its ability to accurately estimate rainfall because manual feature extraction is always biased toward the most relevant and physically obvious features that have direct impacts on precipitation occurrence. Due to the complexity of the precipitation phenomena, there may be some other factors as yet hidden to CHRS researchers that play crucial roles in the accuracy of the model’s simulations. CHRS researchers are currently working on the development of even more advanced satellite retrieval algorithms by applying new data-driven methodologies to automatically extract features from the input datasets^47,48^. While more research needs to be done to verify the performance of the developed dataset, this paper presents promising validation results and demonstrates example applications for the PERSIANN-CCS-CDR dataset. This newly introduced dataset provides an opportunity for scientists and stakeholders to leverage the more accurate estimates of PERSIANN-CCS-CDR to improve disaster mitigation models and strategies.

## Data Availability

MATLAB codes to produce figures and extract data from external databases, as well as the PERSIANN-CCS-CDR algorithm are available in a public GitHub repository (https://github.com/mojtabasadeghi77/PERSIANN-CCS-CDR.git). With any technical difficulties with data downloading and codes, please contact the corresponding author.
